# Supplementary material for: Contribution of p38 MAPK Pathway to Norcantharidin-Induced Programmed Cell Death in Human Oral Squamous Cell Carcinoma
Source: Int J Mol Sci. 2019 Jul 16;20(14):3487. doi: 10.3390/ijms20143487 (PMC6678691; doi:10.3390/ijms20143487)
Supplement: Supplementary file 1 [file ijms-20-03487-s001.pdf]

Supplementary Materials:

Contribution of p38 MAPK pathway to norcantharidin-induced programmed cell death in human oral squamous cell carcinoma

Chi-Hyun Ahn, Kyoung-Ok Hong , Bohwan Jin, WonWoo Lee, Yun Chan Jung , Hakmo Lee, Ji-Ae Shin, Sung-Dae Cho, Seong Doo Hong

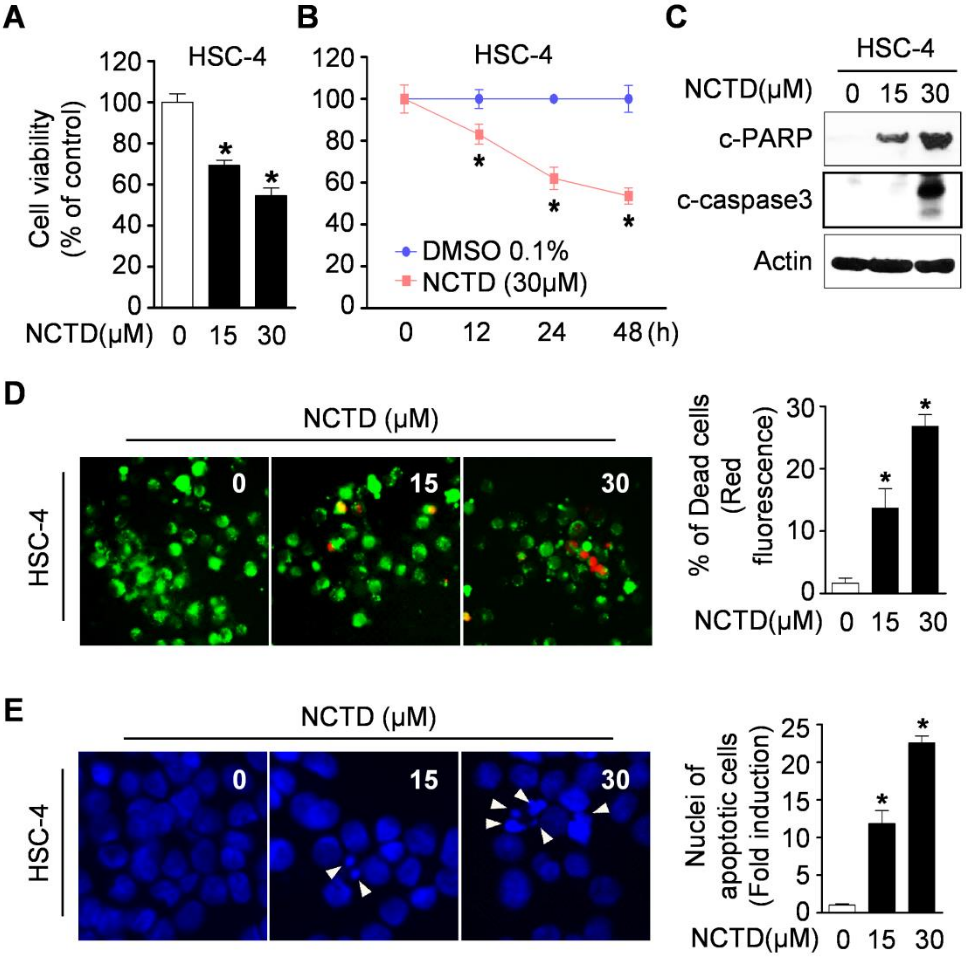

Figure S1. Effects of NCTD on programmed cell death in HSC-4 human OSCC cells. HSC-4 cells were treated with DMSO or certain concentrations of NCTD for 48hr or 30uM NCTD for 12, 24, and 48 hr. (A, B) Cell viability was analyzed using a trypan blue exclusion assay. (C) The cell lysates were analyzed by western blotting to detect the cleavages of caspase 3 and PARP. (D) Cytotoxic effect of NCTD was detected by a live/dead assay kit. The percentage of dead cells was quantified. (E) Nuclear morphology was detected by DAPI staining (indicated by white arrows). Graphs represent the mean ± SD of three independent experiments, and significance compared with the control group is indicated (*).

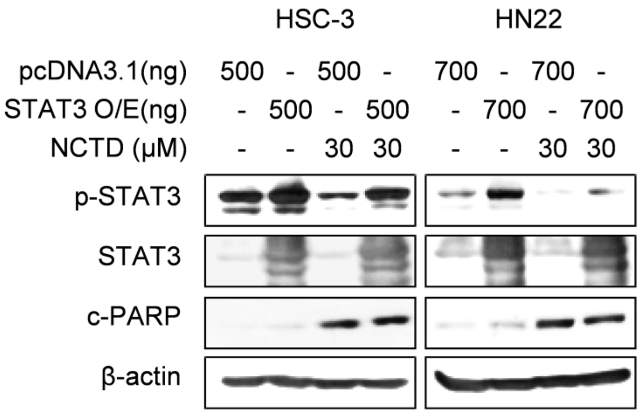

Figure S2. The role of STAT3 on NCTD-induced programmed cell death. HSC-3 and HN22 cells were transiently transfected with pcDNA3.1 or pcDNA3.1-STAT3 for 24hr and treated with 30 μM NCTD for 48hr. The cell lysates were analyzed by western blotting to detect the levels of p-STAT3, STAT3, and cleaved PARP.
